# Minocycline-loaded nHAP/PLGA microspheres for prevention of injury-related corneal angiogenesis

**DOI:** 10.1186/s12951-024-02317-7

**Published:** 2024-03-28

**Authors:** Zitong Li, Wenpeng Huang, Ming Zhang, Yan Huo, Feifei Li, Lele Song, Sitong Wu, Qi Yang, Xiaoming Li, Jianjun Zhang, Liu Yang, Jianchen Hao, Lei Kang

**Affiliations:** 1 Department of Ophthalmology, Peking University First Hospital, Beijing, 100034 People’s Republic of China; 2 Department of Nuclear Medicine, Peking University First Hospital, Beijing, 100034 People’s Republic of China; 3 Department of Pathology, Peking University International Hospital, Beijing, China; 4 Department of Ophthalmology, PLA Rocket Force Characteristic Medical Center, Beijing, China; 5 Beijing Advanced Innovation Center for Soft Matter Science and Engineering, College of Chemical Engineering, Beijing University of Chemical Technology, Beijing, 100029 People’s Republic of China

**Keywords:** Corneal neovascularization, nHAP/PLGA microspheres, Ocular inflammation, Minocycline, Controlled drug release

## Abstract

**Background:**

Corneal neovascularization (CoNV) threatens vision by disrupting corneal avascularity, however, current treatments, including pharmacotherapy and surgery, are hindered by limitations in efficacy and adverse effects. Minocycline, known for its anti-inflammatory properties, could suppress CoNV but faces challenges in effective delivery due to the cornea's unique structure. Therefore, in this study a novel drug delivery system using minocycline-loaded nano-hydroxyapatite/poly (lactic-co-glycolic acid) (nHAP/PLGA) nanoparticles was developed to improve treatment outcomes for CoNV.

**Results:**

Ultra-small nHAP was synthesized using high gravity technology, then encapsulated in PLGA by a double emulsion method to form nHAP/PLGA microspheres, attenuating the acidic by-products of PLGA degradation. The MINO@PLGA nanocomplex, featuring sustained release and permeation properties, demonstrated an efficient delivery system for minocycline that significantly inhibited the CoNV area in an alkali-burn model without exhibiting apparent cytotoxicity. On day 14, the in vivo microscope examination and ex vivo CD31 staining corroborated the inhibition of neovascularization, with the significantly smaller CoNV area (29.40% ± 6.55%) in the MINO@PLGA Tid group (three times daily) than that of the control group (86.81% ± 15.71%), the MINO group (72.42% ± 30.15%), and the PLGA group (86.87% ± 14.94%) (*p* < 0.05). Fluorescein sodium staining show MINO@PLGA treatments, administered once daily (Qd) and three times daily (Tid) demonstrated rapid corneal epithelial healing while the Alkali injury group and the DEX group showed longer healing times (*p* < 0.05). Additionally, compared to the control group, treatments with dexamethasone, MINO, and MINO@PLGA were associated with an increased expression of TGF-β as evidenced by immunofluorescence, while the levels of pro-inflammatory cytokines IL-1β and TNF-α demonstrated a significant decrease following alkali burn. Safety evaluations, including assessments of renal and hepatic biomarkers, along with H&E staining of major organs, revealed no significant cytotoxicity of the MINO@PLGA nanocomplex in vivo.

**Conclusions:**

The novel MINO@PLGA nanocomplex, comprising minocycline-loaded nHAP/PLGA microspheres, has shown a substantial capacity for preventing CoNV. This study confirms the complex's ability to downregulate inflammatory pathways, significantly reducing CoNV with minimal cytotoxicity and high biosafety in vivo. Given these findings, MINO@PLGA stands as a highly promising candidate for ocular conditions characterized by CoNV.

**Graphical Abstract:**

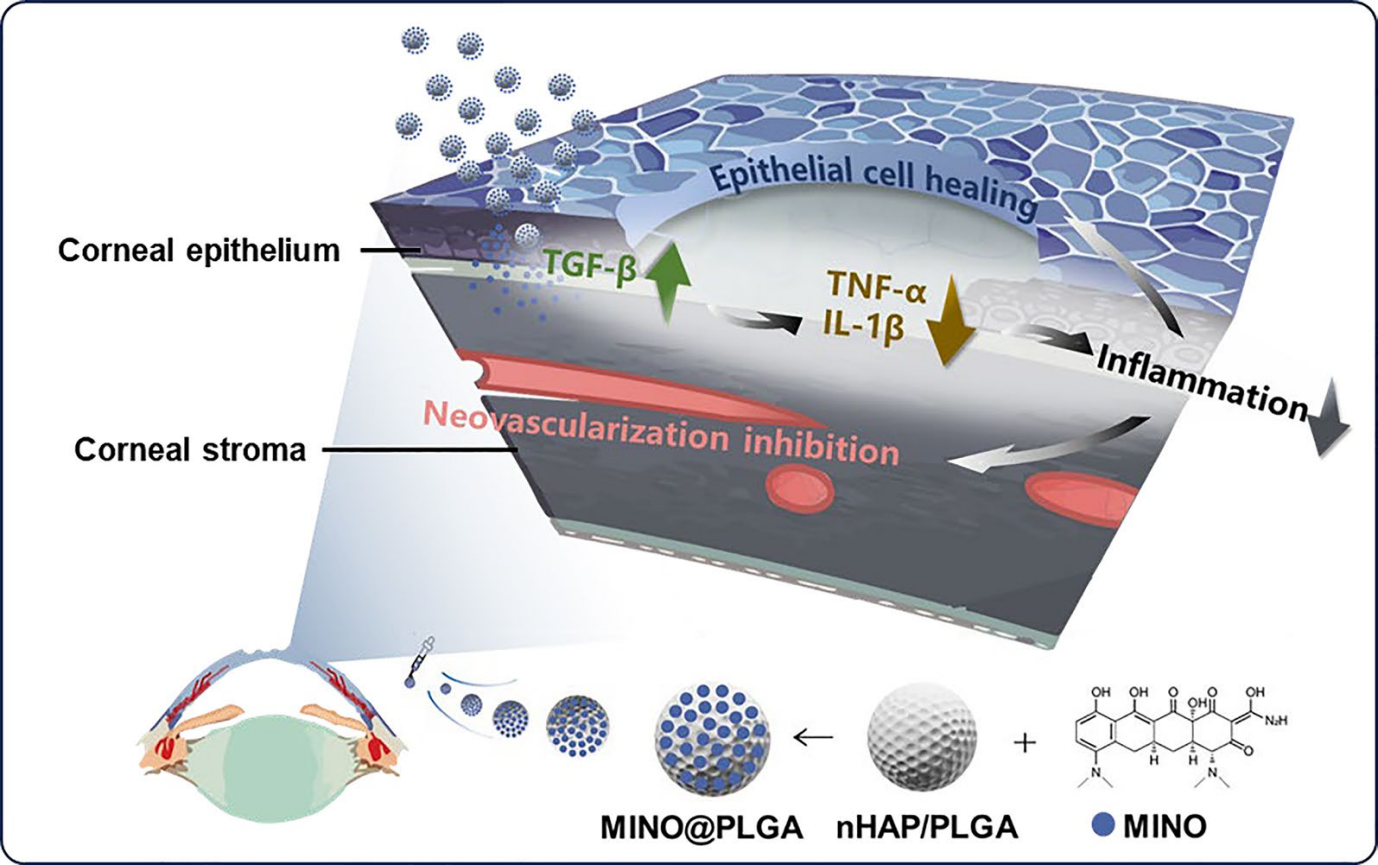

**Supplementary Information:**

The online version contains supplementary material available at 10.1186/s12951-024-02317-7.

## Introduction

Corneal neovascularization (CoNV) as a result of vessel invasion from the limbal arcade to the normally avascular cornea, is a major cause of blindness, afflicting approximately 7 million individuals globally [1]. Several common pathological conditions can lead to CoNV, including infection, trauma, and loss of the limbal stem cell barrier. Normal cornea is transparent and free of blood vessels, and corneal avascularity is critical for optical clarity and maintenance of vision. CoNV leads to decreased corneal transparency, which can induce vision impairment and even lead to blindness [2]. Currently, treatment for CoNV include pharmacotherapy (such as steroids and non-steroidal anti-inflammatory drugs) and surgical interventions (like ocular surface reconstruction) [3]. Clinically, topical corticosteroids are utilized to treat actively proliferating corneal vessels, however, potential adverse reactions may restrict their application. Hence, there's an urgent need for safer and more effective ways to enhance therapeutic outcomes [4].

Minocycline, a type of tetracycline antibiotic, is recognized for its diverse therapeutic properties including anti-inflammatory [5], immunomodulatory [6], and neuroprotective effects [7]. Increasing interest has focused on its anti-inflammatory activities. In the formation process of CoNV, corneal epithelial damage and inflammation play crucial roles. The non-specific anti-inflammatory action of minocycline can inhibit the expression of corneal inflammatory factors, thereby suppressing CoNV. Although intraperitoneal administration of lipophilic minocycline has been shown to be somewhat effective in treating CoNV, the unique structure of the corneal epithelium, which leads to a bioavailability of only about 5%, poses a significant challenge for effective local drug delivery [8].

Currently, research into minocycline eye drops as a treatment option remains scarce. The field of ophthalmology is actively seeking innovative drug delivery systems to circumvent the limitations of conventional therapies [9]. Poly (lactic-co-glycolic acid) (PLGA), a biodegradable aliphatic polyester copolymer, has been approved by the U.S. Food and Drug Administration (FDA) and the European Medicines Agency for various clinical applications, primarily in drug delivery systems [10]. PLGA degrades in aqueous environments through hydrolysis, producing lactic and glycolic acids that are easily metabolized via the Krebs cycle into CO_2_ and H_2_O, facilitating their efficient elimination from the body [11]. In ophthalmology, PLGA has been clinically approved for intraocular implants [12], highlighting its suitability for ocular therapies. However, the use of PLGA, particularly in the form of conventional microspheres, presents specific challenges. One such challenge is the generation of an acidic milieu following degradation, which could potentially affect ocular compatibility [13]. These challenges underscore the need for continued research and innovation in the field of PLGA-based drug delivery systems to enhance their application in ophthalmic treatments. Addressing these issues, our study explores the synergistic potential of combining PLGA with nano-hydroxyapatite (nHAP). nHAP, known for its ability to enhance the adhesion and proliferation of corneal stromal fibroblasts, paving the way for its incorporation into advanced drug delivery systems. In this study, we developed a novel method to enhance therapeutic outcomes, reduce side-effect risks, and achieve high bioavailability of localized drugs. We have formulated biodegradable nanoparticles loaded with minocycline within the nHAP/PLGA composite, designed to maintain a sustained release of the drug. These nanoparticles have the potential to expedite corneal wound healing and attenuate inflammation, thereby improving the treatment outcomes for CoNV and providing a new pharmaceutical option for alkali burns, thus offering valuable insights for future research and clinical applications.

## Materials and methods

### Synthesis and characterization of nHAP

nHAP was fabricated by High gravity technology. Initially, a PASP solution (15 mg/mL) and CaCl_2_ solution (0.1 mol/L) were combined in a volume ratio of 10:1, followed by a chelation process lasting 30 min to create solution A. Concurrently, a PAA solution (25 mg/mL) was mixed with Na_2_HPO_4_·12H_2_O solution (0.06 mol/L) in a 5:1 volume ratio, also chelated for 30 min, forming solution B. These solutions, A and B, were then separately introduced into a Rotating Packed Bed, operating at a specific feed flow rate ratio of 11:12 and a high rotation speed of 2500 rpm/min. This critical step in the process ensured the efficient formation and homogenization of nHAP particles. Finally, to purify and isolate the nHAP, the resultant mixture underwent centrifugation at 8500 rpm/min for 10 min. This was followed by three consecutive washes, resulting in the production of the final nHAP.

Fourier transform infrared (FT-IR) analysis was performed to characterize nHAP. A certain amount of dried nHAP powder was mixed and ground with dried KBr, and the mixture was pressed into pellets using a hydraulic press. The pellets were then tested using a Fourier Transform Infrared spectrometer. X-ray diffraction (XRD) analysis was conducted to determine the crystal structure of nHAP. A certain amount of dried nHAP powder was pressed into glass sample holders and scanned using an X-ray diffractometer at a scanning speed of 5°/min, with the scanning range from 5° to 90°.

### Synthesis and characterization of nHAP/PLGA microspheres

The nHAP/PLGA composite microspheres were fabricated by the double emulsion method. Firstly, dissolving 0.5 g each of PLGA and PDLLA-PEG-PDLLA in 20 mL ethyl acetate, followed by addition of 5 mL nHAP to form a water-in-oil (w/o) emulsion using a homogenizer. This w/o emulsion was then added to a 1% PVA solution to form a water-in-oil-in-water (w/o/w) emulsion. Subsequently, the w/o/w emulsion was vacuumed in a three-neck flask. The resulting solution underwent centrifugation, discarding the supernatant, and washing thrice with ultrapure water. The centrifuged solid was then left overnight in ultrapure water, before undergoing another round of centrifugation and freeze-drying. Finally, 200 mg of the freeze-dried microspheres were washed in a 0.2 mol/L NaOH solution and then in ultrapure water, followed by a final round of freeze-drying, yielding the nHAP/PLGA porous microspheres.

The morphology of the nHAP/PLGA porous microspheres, both pre and post-etching, was characterized using Scanning electron microscopy (SEM). Before freeze-drying, 10 μL of the microsphere suspension was placed on a clean single-polished silicon wafer and allowed to air dry naturally for SEM observation.

### Loading and release of minocycline

5 mg of the microspheres were accurately weighed and dissolved in 1 mL of water, and then uniformly dispersed via ultrasonication. Subsequently, 5 mg of minocycline was added to this dispersed microsphere solution and stirred overnight. After which, the solution was centrifuged at 8000 rpm for 10 min and washed three times with pure water. The supernatant's absorbance at 360 nm was measured and the drug loading capacity of the microspheres was calculated using the standard curve equation. After loading, with a drug loading capacity of approximately 50%, the concentration of minocycline in the microspheres is reduced to 2.5 mg/mL. The microspheres were diluted to 1 mg/mL after loading in the experiment. The concentration of minocycline administered was 0.5 mg/mL.

Precisely weighed drug-loaded microspheres were placed in 5 mL centrifuge tubes. Then, 2 mL of PBS buffer solution was added to each tube. The tubes were incubated in a water bath at 37 °C. At predetermined time intervals (0, 0.25, 0.5, 1, 2, 4, 6, 8, 10, and 14 days), the supernatant was collected after centrifugation, and its absorbance was measured to determine the drug concentration. The drug release percentage was calculated at each time point. The cumulative drug release curve was constructed based on the collected data.

### Degradation of microspheres

Accurately weighed 50 mg of dried microspheres were placed in 10 mL centrifuge tubes, followed by the addition of 5 mL of PBS buffer solution. The samples were dispersed evenly and placed in a 70 °C oven. On days 1, 3, 5, and 7, samples were taken out for analysis. The pH value of the solution was immediately measured using a pH meter.

### CCK-8 assay

Human Umbilical Vein Endothelial Cells (HUVECs) were cultured in ECM medium under conditions of 37 °C and 2% CO_2_. Their cytotoxicity was assessed using the CCK-8 assay. Specifically, HUVECs were seeded in a 96-well plate (1 × 10^4^ cells/well, 100μL/well) and cultured in the medium for 24 h prior to the experiment. Cells were then incubated with MINO at concentrations of 20, 50, 100, 200, or 1000 μg/mL for 24 h. Next, 10 µL of CCK-8 solution was added to each well. After incubation for 2 h in a culture chamber, the absorbance at 450 nm was measured using an ELISA reader to evaluate cell viability. During data analysis, the viability rate of untreated cells was considered as 100%.

### Scratch healing assay

HUVECs were cultured for 24 h under the described conditions. Before the experiment, the cells were serum-starved for 1 h. A scratch was made in the monolayer using a pipette tip, followed by washing to remove debris. The cells were incubated with or without MINO@PLGA and minocycline, and then allowed to migrate for 24 h. Cell migration was observed under a bright-field microscope.

### Animals

Male Sprague–Dawley rats (SD, 180–220 g, 8–9 weeks old) were sourced from Beijing HFK Bioscience Co., Ltd. They were housed at Peking University First Hospital's Animal Center, with all procedures conforming to the ARVO Statement for the Use of Animals in Ophthalmic and Vision Research and approved by the institution's Animal Ethics Committee (Approval No: J202175).

### Induction of corneal neovascularization

Corneal neovascularization was induced via alkali injury as described in previous studies [14], with optimized anesthesia methods [15]. The rats were anesthetized systemically through inhalation of isoflurane (2%, RWD, China). All SD rats’ eyes were routinely examined with a slit lamp before operation to rule out congenital intraocular lesions. The alkali burn was administered by placing a 3 mm diameter filter paper (Whatman 3#, UK) soaked in 1N NaOH onto the central cornea, ensuring full contact for 40 s, followed by immediate rinsing with 20 mL of saline. After the surgery, ofloxacin ointment (Tobrex, Alcon) was applied.

### Treatment

All alkali-burned animals were randomly divided into six groups. These groups included: Control (Alkali injury) group, MINO group, DEX group, PLGA group, MINO@PLGA Qd group, and MINO@PLGA Tid group. The Control (Alkali injury) group received no treatment, the MINO group was treated with minocycline solution (1 mg/mL, 20 µL, three times daily), the DEX group received dexamethasone (1 mg/mL, 20 µL, three times daily), the PLGA group was given empty microspheres (1 mg/mL, 20 µL, three times daily), the MINO@PLGA Qd group received treatment (1 mg/mL, 20 µL, once daily), and the MINO@PLGA Tid group was treated (1 mg/mL, 20 µL, three times daily). Rats in both the treatment and control groups were observed over the following 14 days. Based on the calculated drug loading efficiency and concentration, the maximum daily drug release for the MINO@PLGA Tid group is approximately 0.01 mg.

### Evaluation of CoNV

Corneal neovascularization in post-injury rats was observed and recorded on days 1, 4, 7, and 14 using a microscope, capturing images from five different angles including vertical, superior, inferior, nasal, and temporal positions. Photographic settings were standardized: the microscope magnification was fixed after scale calibration, aperture was set in professional mode, ISO sensitivity was at 50, exposure time was 0.5 s, and white balance was automatically set. The images were analyzed using Image J software. The area of CoNV (A) was quantified using the formula: A = C/12 × π[r^2^(r-l)^2^]. Here, C represents the clock hours covered by corneal NV, l is the average length of the chosen vessels, and r is the radius of the rat's cornea.

Corneal transparency was rigorously evaluated using a systematic approach [16]. The grading of corneal opacity was methodically carried out under microscope examination and classified on a scale from 1 denoting complete transparency to 4, indicating severe opacity. A scoring rubric was applied: 1 for clear corneas, 2 for mild haze, 3 for pronounced opacity occluding iris details, and 4 for intense opacity.

### Corneal fluorescein staining

Fluorescein sodium strips were applied to the cornea to assess epithelial damage, and photographs were taken under the microscope before and on days 0, 1, 2, 3, 4, and 7 post-staining. The staining method involved moistening the strips with 0.1 mL of saline solution, placing it on the rat's lower conjunctival sac, and gently closing the eyelids three times to spread the solution evenly on the corneal surface. Intact corneal epithelium does not stain, while damaged areas appear green in patchy or dotted patterns.

Images were processed using Image-Pro-Plus V6.0 software (Media Cybernetics, USA), and the area of corneal epithelial damage was calculated using the formula: S = S_X_/S_0_ × 100%. Where S_X_ is the stained area observed, and S_0_ is the baseline-stained area.

### Immunostaining

After deparaffinization and rehydration of the tissue sections, antigen retrieval was performed by heating in citrate buffer. Samples were blocked with 10 μL of blocking serum (Solarbio, China, SW3015) at room temperature for 1 h. Slides were incubated overnight at 4 °C with primary antibodies against CD31 (Servicebio, China, 1:200), TGF-β (Abcam, UK, 1:300), IL-1β (Abcam, UK, 1:300), and TNF-α (Servicebio, China, 1:300) Subsequently, they were incubated with IgG-labeled HRP secondary antibody (Servicebio, China, 1:400) at 37 °C for 1 h, followed by staining with DAPI solution (Solarbio, Beijing, S2110) and imaging with a fluorescence microscope (Leica, Heidelberg, Germany, TCS SP2). The mean fluorescence intensity (MFI) was quantified using Image J.

### Confocal microscope

The intrinsic fluorescence intensity of minocycline was observed using a fluorescence microscope, with cell nuclei being labeled with DAPI. MINO@PLGA and minocycline were smeared on slides and calibrated under a fluorescence microscope. Rats were then grouped by various time points and administered the drug once. At the designated time points, rat eyeballs and surrounding tissues were harvested and processed into frozen sections. The frozen sections were fixed in cold acetone for 10 min, then washed, and subsequently mounted using a DAPI medium. Fluorescent images of the cornea were captured using a confocal microscope, employing the specified parameters.

### Biocompatibility

On the 7th day post-alkali eye burn, anesthetize the rat, position it side-eye upwards, and extract 1.5–2.0 mL of blood from the internal canthus orbital venous plexus using a hard glass capillary tube, ensuring it mixes well with anticoagulants. Allow the collected orbital blood to rest at room temperature for 30 min, then centrifuge to obtain the supernatant. Utilize the supernatant in labeled sample cups to run ALT, AST, Cre, and Ure biochemical tests.

After euthanizing the rats, their organs were extracted and then fixed overnight in 4% paraformaldehyde, followed by dehydration and embedding in paraffin. Samples were sectioned to a thickness of 4 mm in preparation for H&E staining. Finally, H&E images were observed under a microscope.

### Statistical analysis

Data were analyzed using one-way ANOVA followed by Bonferroni's multiple comparison test in GraphPad Prism 6.0. Maximum intensity values were presented as mean ± SEM. Differences between means were assessed using the t-test. Statistical significance was set at a p-value of less than 0.05. *p < 0.05, **p < 0.01, ***p < 0.001 or ****p < 0.0001 are marked in the figures.

## Results and discussion

### Characterization of nHAP, nHAP/PLGA, and MINO@PLGA

**Fig. 1:**
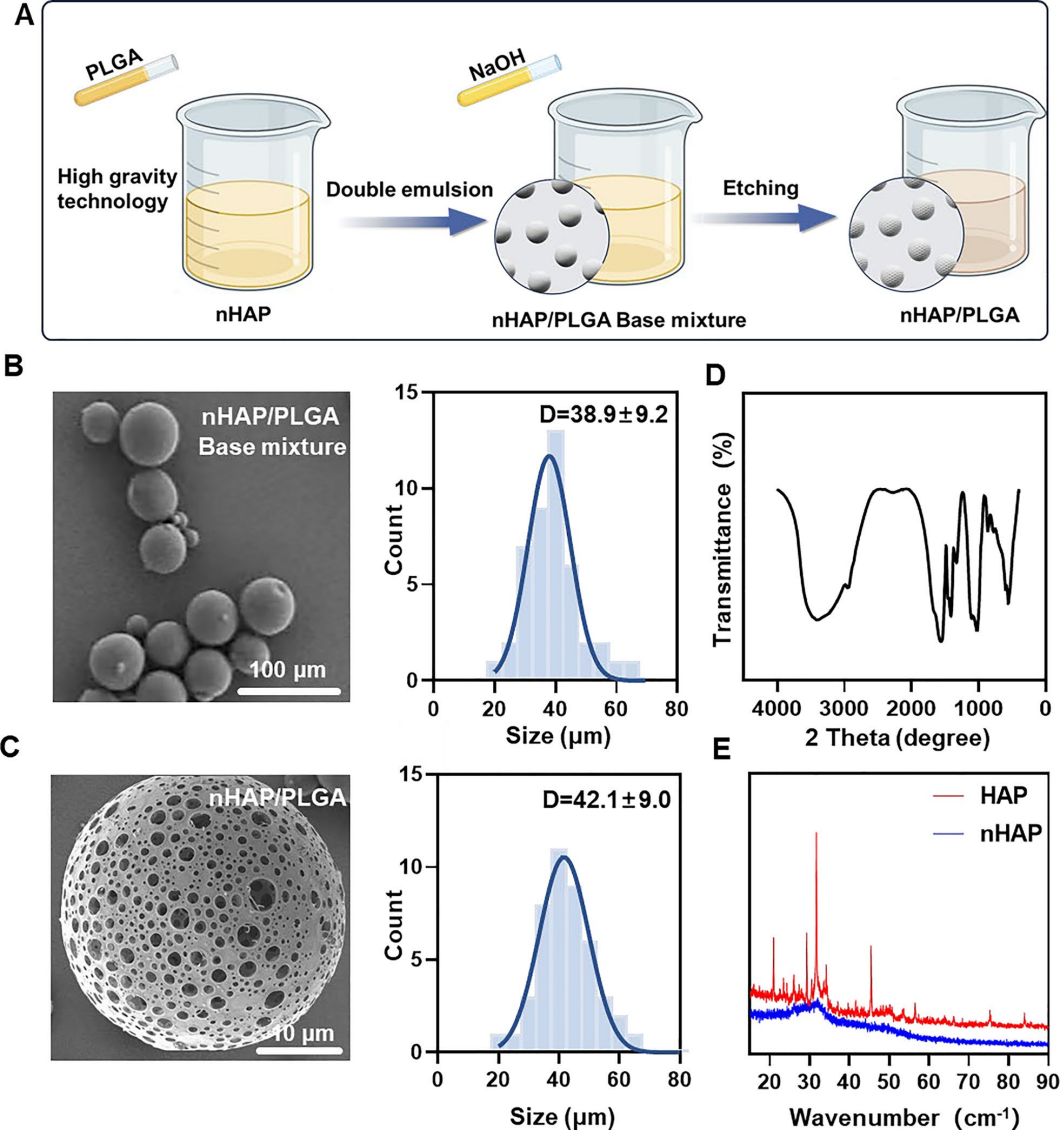
Synthesis and characterizations of nHAP/PLGA. **A** Schematic illustration on the fabrication of nHAP/PLGA. **B**, **C** SEM of nHAP/PLGA base mixture and nHAP/PLGA with the corresponding size of nHAP/PLGA base mixture and nHAP/PLGA. **D** FT-IR characterization of nHAP. **E** XRD characterization of nHAP and HAP

MINO was loaded onto porous nHAP/PLGA microspheres in a simple, efficient, cost-effective, and environmentally-friendly manner to synthesize MINO@PLGA. It functions by enhancing TGF-β expression, reducing ocular inflammation, accelerating corneal epithelial healing, and inhibiting neovascularization (Graphical Abstract). Initially, a nano-hydroxyapatite (nHAP) with ultra-small size and high dispersion stability is prepared using ultracentrifugation technology followed by combining it with PLGA to form a base mixture. This mixture is then etched with NaOH to produce the final nHAP/PLGA microspheres (Fig. 1A). SEM reveals that nHAP/PLGA base mixture has a good dispersibility and spherical shape with particle size of 38.9 ± 9.2 μm (Fig. 1B). In contrast, the etched nHAP/PLGA microspheres exhibit a porous structure suitable for loading MINO with particle size of 42.1 ± 9.0 μm (Fig. 1C). The FT-IR spectrum of nHAP (Fig. 1D), displays peaks for O–H at 3300 cm^−1^ to 3400 cm^−1^ and 1500 cm^−1^ to 1600 cm^−1^. Peaks for P-O are evident at 1000 cm^−1^ to 1100 cm^−1^ and 500 cm^−1^ to 600 cm^−1^. This indicates that the product is hydroxyapatite. X-ray diffraction (XRD) indicates that nHAP has lower diffraction peaks compared to HAP, suggesting enhanced crystalline stability in the synthesized nHAP (Fig. 1E).

Incorporating nHAP retains the scaffold's biocompatibility and improves the biodegradability of the microspheres. Moreover, the XRD of nHAP displays an amorphous structure, indicating enhanced drug loading and sustained-release capabilities. The drug-loading capacity of nHAP/PLGA microspheres, measured at an absorbance of 360 nm, is approximately 50%. Biodegradable polymers have garnered increasing interest due to their biocompatibility, non-toxicity, and diverse properties. PLGA stands out as one of the most widely recognized biodegradable polymers. Notably, it has received approval from the FDA for applications in drug delivery, diagnostics, and various fields of clinical and basic science research, encompassing cardiovascular disease, cancer, vaccines, and tissue engineering [17]. An in vivo ocular anti-inflammatory study conducted in rabbit eyes confirmed the superior efficacy of drug-loaded nanoparticles in comparison to drug solutions [18]. Two research teams have also designed PLGA nanoparticles loaded with tacrolimus for topical ocular instillations [19]. Alshamsan et al. developed PLGA-tacrolimus nanoparticles using the emulsification-diffusion method, demonstrating a significant enhancement in ocular bioavailability through the entrapment of tacrolimus by nanoparticles [20]. Similarly, Benita et al. developed PLGA-tacrolimus nanoparticles using a well-established solvent displacement method. It's worth noting that repeated ocular instillations of these nanoparticles in rat eyes resulted in elevated tacrolimus levels within the eye, while plasma concentrations remained low [19].

Furthermore, the size of micro-particles plays a crucial role in the drug release rate. A reduction in particle size leads to an increase in surface area and mass transfer for a fixed mass of drug and polymer [21]. Lyu et al. [9] encapsulated bevacizumab (BEV) within the pores of mesoporous silica nanoparticles (MSNs), forming BEV@MSN nanoparticles with an average diameter of 39.3 ± 5.3 nm. Meanwhile, Zhang et al. [22] prepared PLGA-bevacizumab nanoparticles with a hydrodynamic diameter of approximately 133 nm, achieving an entrapment efficiency and loading efficiency of around 80.0% and 6.8%, respectively. In this study, the loading of MINO into porous nHAP/PLGA microspheres, with a drug loading capacity of approximately 50%, could enhance the bioavailability and augment the anti-angiogenic efficiency of MINO. Moreover, MINO@PLGA nanoparticles exhibit a spherical shape with a smooth surface, which could minimize susceptibility to shear forces and facilitate effective interactions with cell surfaces, thereby increasing cellular uptake. Consequently, PLGA-mediated MINO delivery promises to extend the residence time of MINO in vivo, leading to sustained concentrations and improved drug bioavailability.

### MINO@PLGA microspheres release, degradation, permeation and inhibit cell migration

**Fig. 2:**
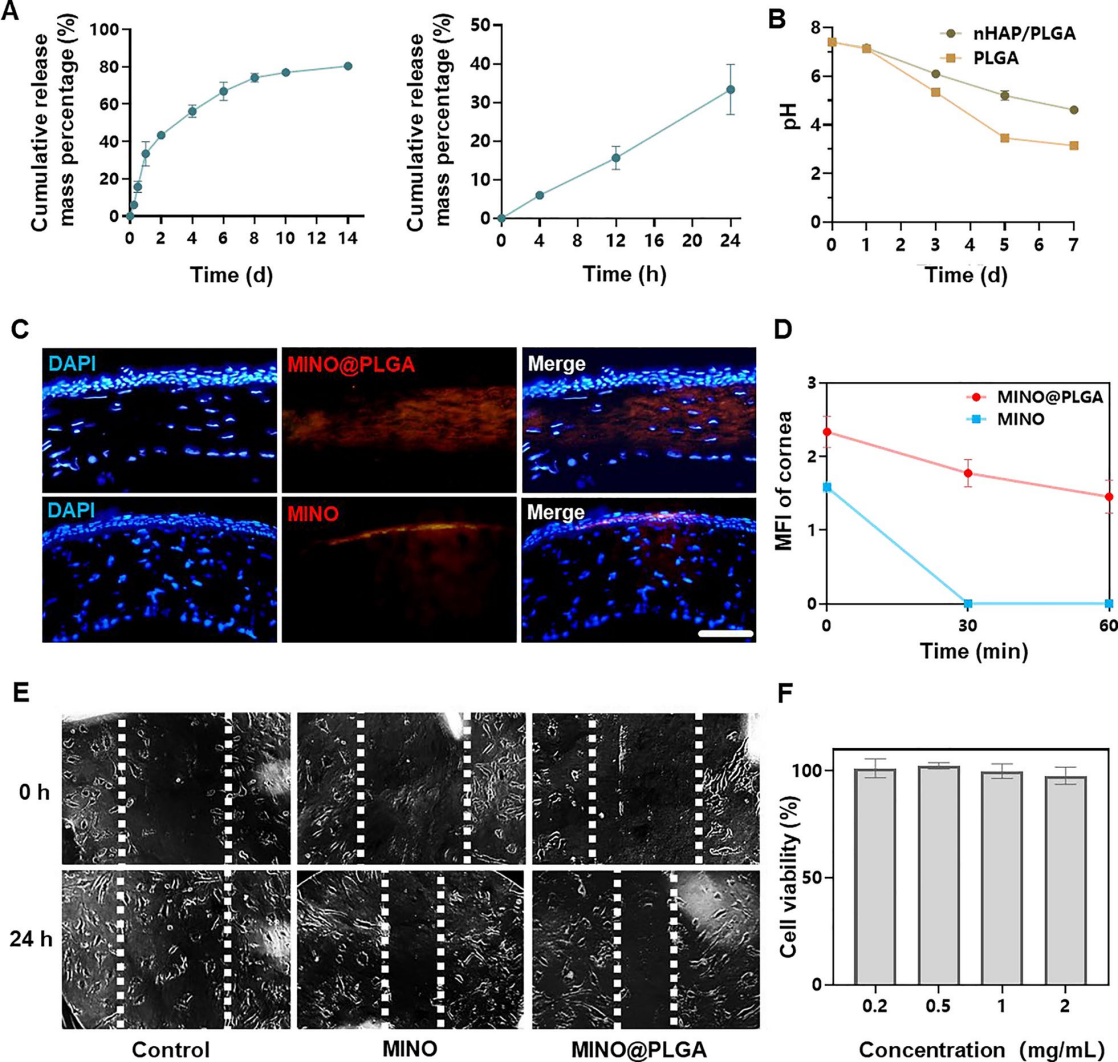
Minocycline microspheres release, degradation, permeation and inhibit cell migration. **A** Minocycline microspheres release profile. **B** Degradation of nHAP/PLGA over time with pH. **C** Fluorescent retention observation of e minocycline or MINO@PLGA in cornea (scale bar: 100 μm). **D** Changes in the cornea average fluorescence intensity of drug after eye drop at 0, 30, and 60 min (n = 3). **E** Scratch healing assay of HUVECs treated with minocycline (1 mg/mL) or MINO@PLGA (1 mg/mL). Representative images of the scratch gap were captured at 0 h and 24 h. **F** Effect of MINO@PLGA on viability of HUVECs

The cumulative release of MINO from PLGA microspheres increased over time, reaching approximately 80% by day 14 (Fig. 2A). This sustained release pattern suggests that the microspheres provide a prolonged therapeutic effect, potentially countering the activity of corneal neovascularization over an extended period. The pH of PLGA microspheres declined within 7 days, indicating the degradation of PLGA microspheres might result in an acidic environment (Fig. 2B). However, due to the irritant nature of low pH on eyes, there are limitations to the use of PLGA microspheres. In contrast, nHAP/PLGA demonstrated a more stable pH post-degradation compared to PLGA, suggesting better tissue compatibility. Combined images revealed that MINO@PLGA effectively remained within the corneal epithelium, whereas standalone MINO failed to penetrate the corneal epithelium (Fig. 2C). This indicates that MINO@PLGA enhances drug contact time with ocular tissues, providing a controlled and protected delivery of drugs, potentially extending therapeutic effects. Pharmacokinetic data derived from fluorescence intensity measurements of the cornea (Fig. 2D) provide the sustained release profile of our formulation. At the 60-min mark, a significant difference in fluorescence intensity was observed between the MINO and MINO@PLGA groups. This difference indicates that the MINO@PLGA microspheres not only facilitate enhanced corneal penetration but also contribute to a prolonged drug contact time with ocular tissues. Specifically, while the average retention time of standard ocular medications on the eye surface is 2–3 min [23], our MINO@PLGA formulation exhibited prolonged residence time. Such data underscore the efficacy of the MINO@PLGA system in maintaining sustained drug concentrations at the target site, thus potentially extending therapeutic effects.

This suggests that MINO@PLGA microspheres, with their sustained release and enhanced corneal penetration, contribute to prolonged drug contact with ocular tissues. Both MINO and MINO@PLGA exhibited inhibitory effects on HUVEC migration after 24 h, with MINO@PLGA demonstrating a slightly stronger effect (Fig. 2E, F). This indicates that encapsulated MINO retains its bioactivity, and the sustained release from PLGA microspheres may enhance its inhibitory effects, making it a potential superior agent for wound healing or anti-angiogenic applications.

The severity of corneal alkali burns and the extent of tissue damage can significantly impact the timing and intensity of the inflammatory response. In certain instances, inflammation and CoNV may persist for an extended duration, particularly when the injury is severe or complicated by issues such as infection. The majority of current treatments exhibit optimal efficacy within a relatively limited temporal window following the initiation of CoNV, with diminishing effectiveness as neovascularization matures and stabilizes [2]. The BEV@MSN nanoparticles, as prepared by Lyu et al. [9], exhibited a release duration of up to four weeks, with only about 27% and 33% released on the first and second days, respectively, followed by a nearly linear release over the subsequent three weeks. In contrast, the PLGA-bevacizumab nanoparticles prepared by Zhang et al. [22] demonstrated a different release profile, with more than 40% of bevacizumab being released within the first 2 h, and an additional approximate 40% released in the subsequent 7 days, followed by a slower release extending up to 21 days. MINO@PLGA extends the half-life of MINO and achieves sustained release from PLGA nanoparticles in a stable, controlled manner. This sustained release has the potential to continuously counteract the activity of corneal neovascularization. MINO@PLGA retains more effectively in the cornea, prolonging drug contact time with ocular tissues, delivering drugs to a specific tissue site in a controlled manner, protecting drugs from degradation. This may be attributed to the good water solubility of the MINO@PLGA microspheres and the superior liposolubility of MINO within the tetracycline category, enabling it to penetrate the corneal epithelium and reach the corneal stroma [24].

Abnormal endothelial cell proliferation, migration, and tube formation is critical for angiogenic effects [25]. MINO@PLGA might exhibit a stronger inhibitory effect on HUVECs migration, potentially making it a superior agent for wound healing or anti-angiogenic applications [26]. In addition, inadequate corneal penetration and the rapid clearance of drugs from the ocular surface significantly undermine the therapeutic efficacy of topical eye drops for the treatment of ocular diseases. Consequently, frequent administration of topical drugs or high-dose systemic medication is often required (30–60 mg/kg MINO daily) [15], which may result in reduced patient compliance and compromised therapeutic effectiveness. In our study, the dose and frequency of drug administration could be decreased.

### Evaluation of anti-CoNV effect

**Fig. 3:**
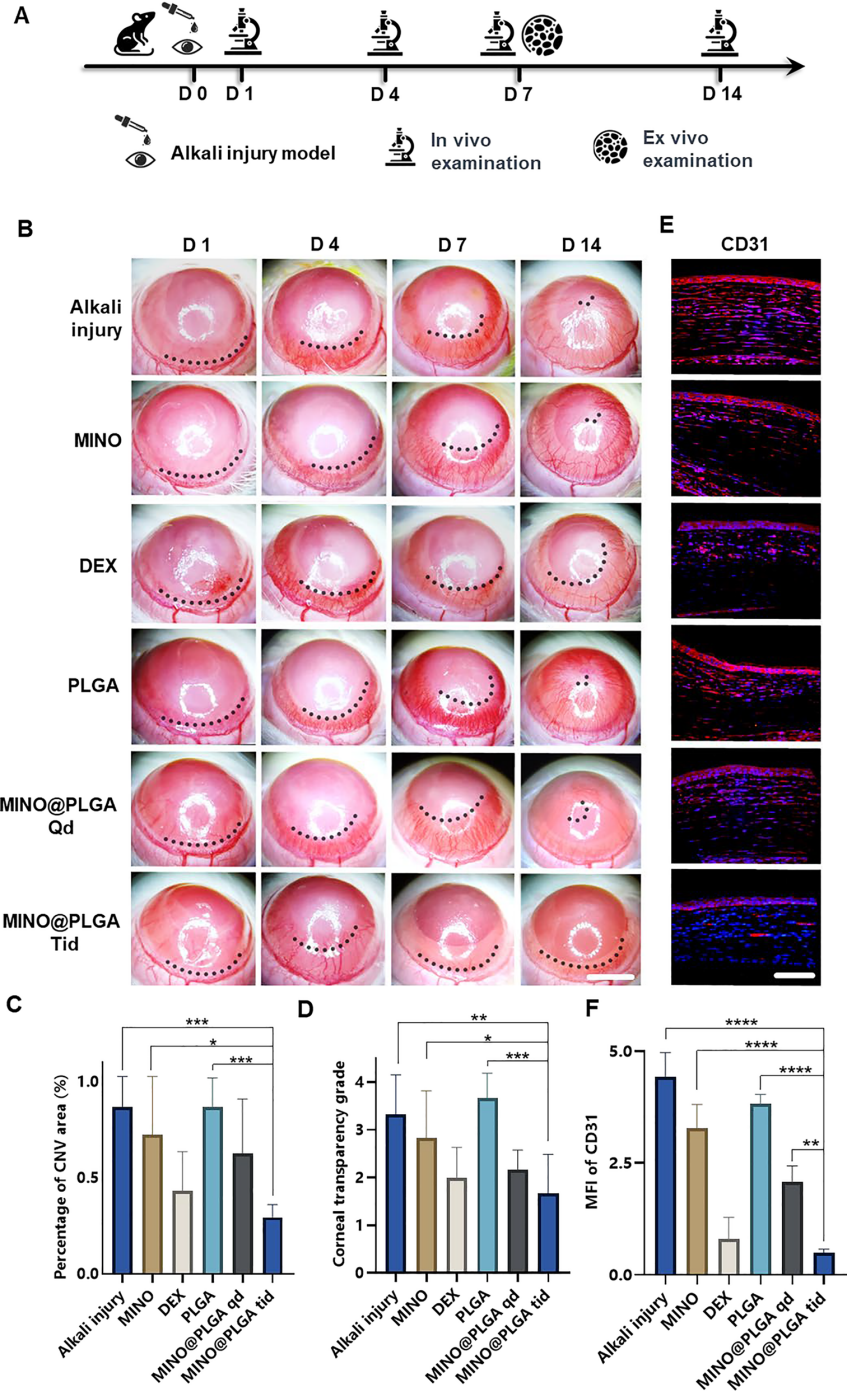
MINO@PLGA inhibits corneal neovascularization and reduces haze. **A** Examination time flow after corneal alkali burn. **B** The corneal neovascularization was examined at 1, 4, 7, 14 days after the corneal alkali burn (scale bar: 3 mm), and the quantified corneal neovascularization of 14 days is shown in (**C**) (n = 6). **D** Corneal opacity at 14 days between groups (n = 6). **E** CD31 immunofluorescence at 7 days. **F** MFI of CD31 (scale bar: 100 μm, n = 3). *p < 0.05; **p < 0.01; ***p < 0.001; ****p < 0.0001

The corneal alkali burn model was established on day 0, followed by continuous observation for 14 days post-operation. Slit-lamp in vivo examinations were conducted on days 1, 4, 7, and 14, with a subset of rats sacrificed for ex vivo analysis on day 7 (Fig. 3A). The inhibitory effect of MINO@PLGA on CoNV was evaluated by continuously observing and comparing the CoNV area percentage and corneal transparency at the same corneal location between the MINO@PLGA (Qd and Tid) groups and the control group. From day 1 and 4 post-alkali burn, the neovascular buds in the MINO@PLGA (Qd and Tid) groups showed similarity to those in the control group (*p* > 0.05). From day 4 to day 14, corneal neovascularization in the group treated with MINO@PLGA Tid exhibited a decrease. In contrast, CoNV growth in the control group was notably robust, and it is time-dependent (Fig. 3B). The neovascular area ratio was further quantified. As shown in Fig. 3C, on day 14, the CoNV area for the MINO@PLGA Tid group was the smallest at 29.40% ± 6.55%. These values were lower than those in the control group (86.81% ± 15.71%), MINO group (72.42% ± 30.15%), PLGA group (86.87% ± 14.94%) (*p* < 0.05), and the DEX group (43.23% ± 20.35%), MINO@PLGA Qd group (62.78% ± 28.12%) (*p* > 0.05). Quantification confirmed the visual observations; the reduction of CoNV in corneas treated with MINO@PLGA suggests its potential in effectively managing post-injury corneal neovascularization. Corneal transparency, reflecting the edematous state, injury, and inflammatory condition, varied among treatments. MINO@PLGA (Qd and Tid) treatments led to decreased opacity (*p* < 0.05), suggesting improved wound healing and attenuation of inflammation crucial for maintaining corneal clarity (Fig. 3D). Samples treated with MINO@PLGA (Qd and Tid) exhibited decreased CD31 fluorescence, indicating reduced vascular formation (Fig. 3E). Compared to other samples (except for the DEX group), those treated with MINO@PLGA, especially Tid, demonstrated a notably lower MFI of CD31 (Fig. 3F) (*p* < 0.05).

Minocycline, a tetracycline derivative, possesses the ability to penetrate the blood–brain barrier and serves as a broad-spectrum antibiotic with demonstrated anti-inflammatory, anti-apoptotic, and antioxidant properties [27]. Its extensive usage includes the treatment of respiratory infections, reproductive tract infections, skin infections, and suppurative infections [28]. Our previous study demonstrated minocycline has neuroprotective effect via its anti-inflammation mechanism [7]. Furthering the understanding of minocycline's therapeutic effects, research by Yuan et al. [29] revealed its significant anti-inflammatory activity in rat models of endotoxin-induced uveitis and retinal inflammation. This was achieved primarily through the attenuation of LPS-induced expression of IL-1β and CCL-2. Previous research [15] involving systemic administration of minocycline, particularly through intraperitoneal injection in a mouse model of alkali burn-induced corneal neovascularization, demonstrated a decrease in CoNV to 73.03% ± 17.81%, in stark contrast to 97.43% ± 3.91% observed in the PBS group on day 14. In our study, we observed a markedly more significant reduction in the CoNV area with the MINO@PLGA treatment—specifically, 29.40% ± 6.55% compared to 86.81% ± 15.71% in the control group on day 14. This finding not only underscores the effectiveness of our MINO@PLGA treatment in reducing CoNV but also extends previous observations by showing that minocycline, when delivered effectively, can substantially decrease the extent of CoNV. These results solidify minocycline's potential as a valuable therapeutic agent in the treatment of CoNV.

The reduced opacity with MINO@PLGA treatment suggests improved wound healing and attenuation of inflammation, which is crucial for maintaining corneal clarity [30]. The lower MFI of CD31 in MINO@PLGA-treated samples further quantifies its anti-angiogenic properties [31], suggesting a potential similar to the anti-inflammatory drug dexamethasone in managing corneal neovascular diseases, which was superior to MINO alone [32]. And Compared to the MINO@PLGA Qd group, the MINO@PLGA Tid group demonstrates the ability to maintain a sufficient drug concentration, thus improving efficacy in the treatment of CoNV and reducing opacity.

### Injury healing

**Fig. 4:**
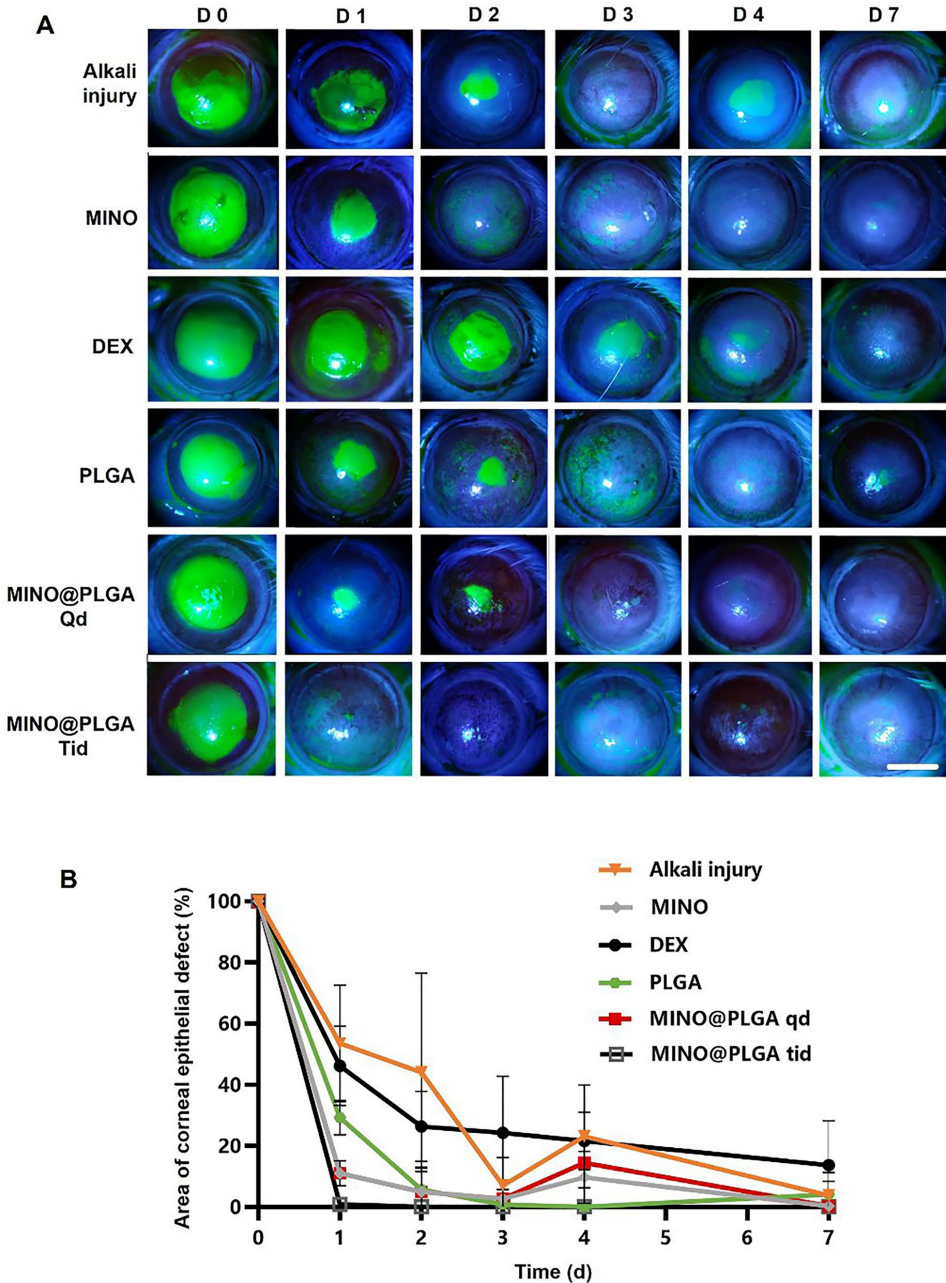
Changes in the corneal epithelial injury area over time in different groups after alkali burn. **A** Typical fluorescein sodium staining of the corneal epithelium was examined at 0, 1, 2,3, 4, 7 days after the corneal alkali burn (scale bar: 3 mm), the percentage of corneal epithelial defect area over time is shown in (**B**) (n = 6)

Fluorescein sodium staining was employed to visualize the progression of corneal epithelial injuries across all groups (Fig. 4A). Notably, both the MINO group and the MINO@PLGA group exhibited no signs of nonhealing in corneal epithelial injury repair, highlighting the efficacy of MINO and MINO@PLGA in promoting damage repair and controlling inflammation. Figure 4B illustrates the comparative analysis of epithelial healing rates among different treatment groups. MINO@PLGA (Qd and Tid) demonstrated a notably faster wound healing rate and better epithelial function recovery compared to other treatments, including the DEX group. The MINO@PLGA Tid group, in particular, exhibited efficient promotion of epithelial healing post-alkali burn. Corneas treated with MINO@PLGA Tid demonstrated rapid reductions in epithelial defect areas, with almost complete healing observed by day 7. In contrast, the Alkali injury group and the DEX group showed slower corneal epithelial healing and longer healing times (*p* < 0.05).

Corneal keratolysis, often associated with heightened inflammation and impaired re-epithelialization, necessitates strategies that minimize inflammation and expedite re-epithelization to prevent complications [33, 34]. It is known that glucocorticoids, such as dexamethasone (DEX), can impede wound healing by hindering cell migration, as demonstrated in previous studies [35, 36]. Our findings align with this notion, showing that the DEX group exhibited delayed corneal epithelial healing compared to other treatments.

In contrast, both the MINO group and the MINO@PLGA group displayed efficient corneal epithelial injury repair, underscoring the advantages of MINO and MINO@PLGA in promoting damage repair while effectively controlling inflammation. Fluorescein sodium staining confirmed the progress of corneal epithelial injuries, providing a visual representation of the positive outcomes associated with MINO and MINO@PLGA treatments.

The results from Fig. 4B further emphasize the superiority of MINO@PLGA (Qd and Tid) in promoting epithelial healing compared to other treatments, including the DEX group. The faster recovery observed in the MINO@PLGA Tid group suggests that frequent application of this formulation may offer superior treatment effects in promoting wound healing after alkali injury. This aligns with the crucial role of accelerated re-epithelialization in advancing overall wound healing [37]. These findings collectively highlight the potential clinical significance of MINO@PLGA in managing acute ocular alkali burns by facilitating prompt corneal epithelial healing while minimizing inflammation-related complications.

### Immunofluorescent staining

**Fig. 5:**
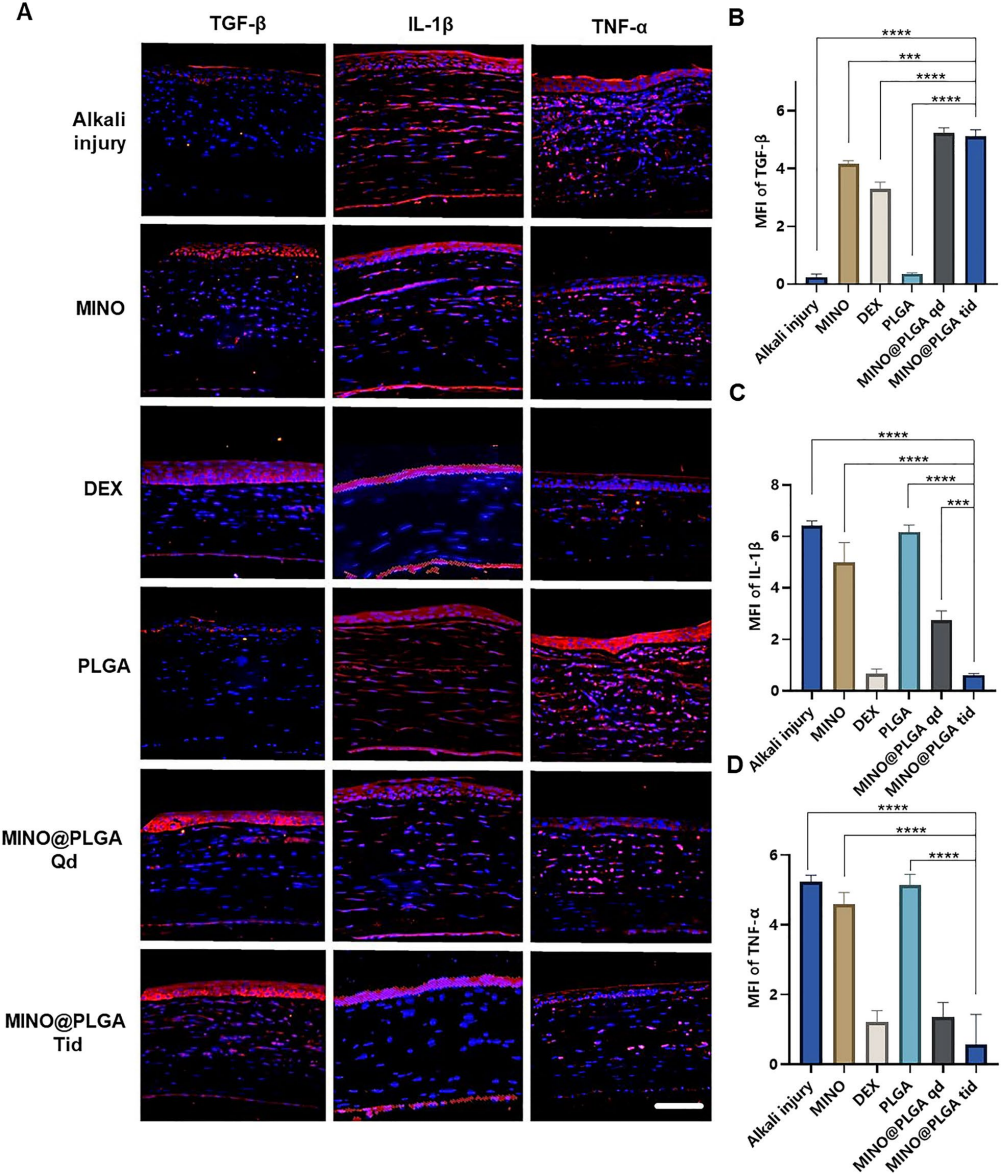
Immunofluorescence. **A** Typical IF picture of TGF-β, IL-1β and TNF-α in cornea (scale bar: 100 μm). Average fluorescence intensities of corneal TGF-β (**B**), IL-1β (**C**), and TNF-α (**D**) at 7 days after alkali burn in different groups (n = 3). ***p < 0.001; ****p < 0.0001

Images reveal different levels of corneal TGF-β and inflammatory factors IL-1β and TNF-α in various treatment groups on day 7 post-alkali burn. In the control group, the expression level of TGF-β in the corneal epithelium is decreased, while levels of IL-1β and TNF-α in the corneal stroma are elevated. With dexamethasone, MINO, and MINO@PLGA treatments, there is an increased expression of TGF-β, while inflammatory factors IL-1β and TNF-α show varying degrees of decrease (Fig. 5A). Semi-quantitative analysis results (Fig. 5B) indicate that the expression of TGF-β was highest in the MINO@PLGA Qd and MINO@PLGA Tid groups (5.23 ± 0.18, 5.12 ± 0.23 respectively), followed by the MIMO group (4.16 ± 0.12). The Dexamethasone group showed a slight decrease (3.23 ± 0.25), while the Alkali injury and PLGA groups exhibited the lowest values (0.24 ± 0.10 and 0.35 ± 0.03 respectively). The expression of IL-1β and TNF-α was lowest in the MINO@PLGA Tid group (0.61 ± 0.04 and 0.57 ± 0.50, respectively) and showed statistically significant differences when compared to the control group (*p* < 0.0001) (Fig. 5C, D). The reduction of inflammatory in the cornea was also evidenced by H&E staining of corneal tissue (Additional file 1: Fig. S1). This suggests that MINO@PLGA might suppress CoNV through the downregulation of inflammatory factors IL-1β and TNF-α via the TGF-β pathway.

During alkali burns, the corneal epithelium sustains damage, resulting in a deficiency of pluripotent limbal stem cells and an imbalance between angiogenic stimulators and inhibitors. This imbalance can lead to the over-proliferation and migration of capillary endothelial cells into the damaged cornea [38]. The corneal epithelium plays a pivotal role in the mechanism of neovascularization. The expression of inflammatory factors induced by TGF-β exerts anti-neovascular effects [39]. TGF-β has also been reported to stimulate the transdifferentiation of corneal stromal cells into myofibroblasts, thereby increasing corneal opacity [40]. Pruzanski et al. [41] demonstrated that minocycline inhibits phospholipase A2 (PLA2) and interacts with the substrate to attenuate the inflammatory response in patients with rheumatoid arthritis. In the context of inflammation, glial cells, both astrocytes and microglia, play pivotal roles [42]. Recent studies have shown that minocycline mitigates the development of pain hypersensitivity by inhibiting microglial activation, reducing proinflammatory cytokine expression in both inflammatory and neuropathic pain, and downregulating the production of proinflammatory cytokines, such as TNF-α, IL-1β, and IL-6 [43]. Furthermore, minocycline was found to inhibit p38 MAPK in microglia, specifically targeting this pathway. P38 MAPK is known to mediate inflammatory responses in various cell types, including microglia.

With the progressive elucidation of minocycline's anti-inflammatory effects and underlying mechanisms, numerous studies have demonstrated its multifaceted properties in various animal models of neuronal degenerative diseases. Minocycline exhibits antimicrobial, anti-inflammatory, anti-apoptotic, and neuroprotective characteristics in conditions such as Parkinson's disease, multiple sclerosis, Alzheimer's disease, Huntington's disease, amyotrophic lateral sclerosis, and retinitis pigmentosa [44, 45]. Scholz et al. [46] demonstrated that minocycline provides protection against retinal damage in a mouse model of acute retinal degeneration induced by white-light irradiation. Administration of minocycline led to the inhibition of inflammatory factors, including CCL2, IL-6, and iNOS, and resulted in the suppression of pro-inflammatory responses and neurotoxicity in microglia, while promoting the survival of photoreceptors. In this study, we have demonstrated, for the first time, that minocycline, via the TGF-β pathway, downregulates the inflammatory factors IL-1β and TNF-α to effectively suppress CoNV.

### Biocompatibility

**Fig. 6:**
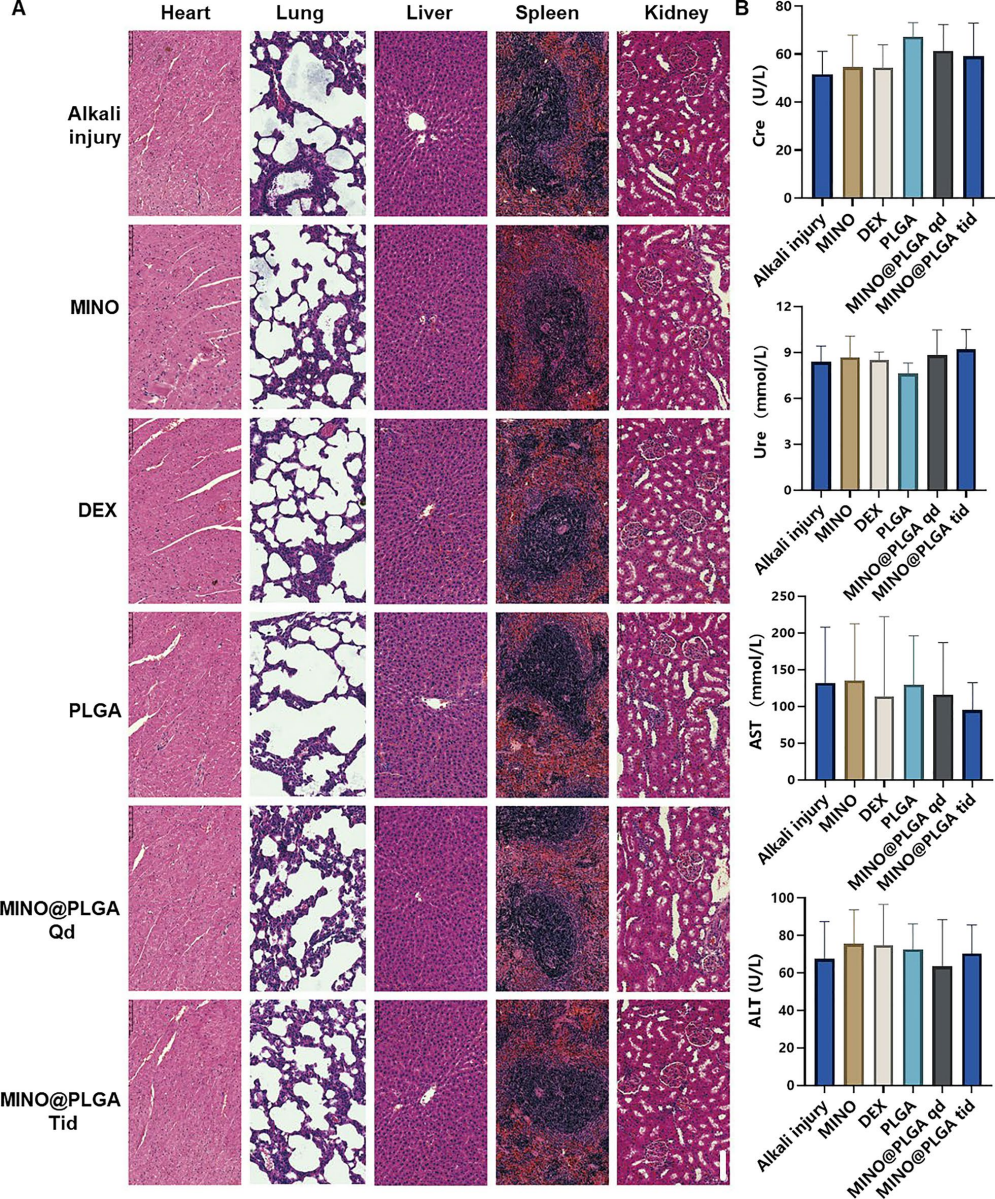
Evaluation of MINO@PLGA safety on organ histopathology and systemic blood parameters. **A** H&E of major organs in rats from different groups at 7 days after corneal alkali burn (scale bar: 100 μm). **B** Biochemical indicators in the orbital venous blood of rats from different groups at 7 days after alkali burn. Creatinine (Cre) reference range: 19.43–64.97 mmol/L. Urea (Ure) reference range: 2.08–7.75 mmol/L. Alanine aminotransferase (ALT) reference range: 33.7–98.7 U/L. Aspartate aminotransferase (AST) reference range: 69.7–322.9 U/L (n = 6)

To assess the biosafety of MINO@PLGA, histological examination of major organ tissues (Heart, lung, liver, spleen, kidney) was performed using H&E staining (Fig. 6A). The images across all treatment groups, including MINO@PLGA treatments, revealed no overt signs of organ damage. This suggests a favorable safety profile for MINO@PLGA, with no discernible pathological changes in major organs following its use post-alkali burn. Biochemical parameters from blood samples, including Creatinine (Cre), Urea (Ure), Alanine aminotransferase (ALT), and Aspartate aminotransferase (AST), were analyzed to further evaluate the safety of MINO@PLGA (Fig. 6B). The data indicated that these parameters remained within normal reference ranges across all treatment groups. The maintenance of normal levels suggests that MINO@PLGA does not adversely impact renal or liver function, reinforcing its safety as a treatment option following alkali burns.

The systemic administration of minocycline is associated with various common adverse side effects [47]. Serious adverse effects include hypersensitivity syndrome reaction, drug-induced lupus, idiopathic intracranial hypertension, and other autoimmune syndromes, which may lead to fatal outcomes [43]. To enhance therapeutic efficacy and achieve highly localized effects while minimizing the risk of side effects compared to systemic administration, the use of locally delivered drug-loaded biodegradable nanoparticles presents an appealing alternative [6]. Topical administration, necessitating a significantly lower dosage than systemic routes, is favored for ophthalmic drug delivery [48]. Local injections, such as subconjunctival or intravitreal, are invasive and thus reserved for administration by healthcare professionals. Our study emphasized the topical administration of eye drops, a patient-friendly method that can be self-administered [49].

## Conclusion

In this study, a novel nanocomplex was developed by loading minocycline onto porous nHAP/PLGA microspheres, effectively inhibiting corneal neovascularization through the regulation of inflammatory pathways, including the upregulation of TGF-β and the downregulation of IL-1β and TNF-α. The MINO@PLGA eye drop therapy demonstrated minimal cytotoxicity and high in vivo biosafety. Furthermore, the nanohybrid exhibited the most potent in vivo therapeutic inhibition of corneal neovascularization, including reduced CoNV area, corneal haze, and inflammation. Consequently, this developed nanohybrid represents a promising alternative for the treatment of ocular diseases associated with corneal neovascularization.

### Supplementary Information

**Additional file 1: Figure S1.** Appearance of the corneal structures by H&E.

## Data Availability

Not applicable.

## Abbreviations

CoNV: Corneal neovascularization
nHAP: Nano-hydroxyapatite
PLGA: Poly (lactic-co-glycolic acid)
nHAP/PLGA: Nano-hydroxyapatite/poly (lactic-co-glycolic acid)
MINO: Minocycline
MINO@PLGA: Minocycline-loaded nHAP/PLGA
Tid: Three times daily
Qd: Once daily
D: Day
DEX: Dexamethasone
PASP: Polyaspartic acid
CaCl_2_: Calcium chloride
PAA: Polyacrylic acid
Na_2_HPO_4_·12H_2_O: Sodium phosphate dodecahydrate
FT-IR: Fourier transform infrared
XRD: X-ray diffraction
SEM: Scanning electron microscopy
H&E: Hematoxylin and eosin
SD: Sprague–Dawley
PBS: Phosphate-buffered saline
SD: Standard deviation
ANOVA: One-way analysis of variance
BEV: Bevacizumab
MSN: Mesoporous silica nanoparticles
MFI: Mean fluorescence intensity
Cre: Creatinine
Ure: Urea
AST: Aspartate aminotransferase
ALT: Alanine aminotransferase
