# Supplementary figures and images for: Minocycline-loaded nHAP/PLGA microspheres for prevention of injury-related corneal angiogenesis
Source: J Nanobiotechnology. 2024 Mar 28;22:134. doi: 10.1186/s12951-024-02317-7 (PMC10979583; doi:10.1186/s12951-024-02317-7)

## Slide 1
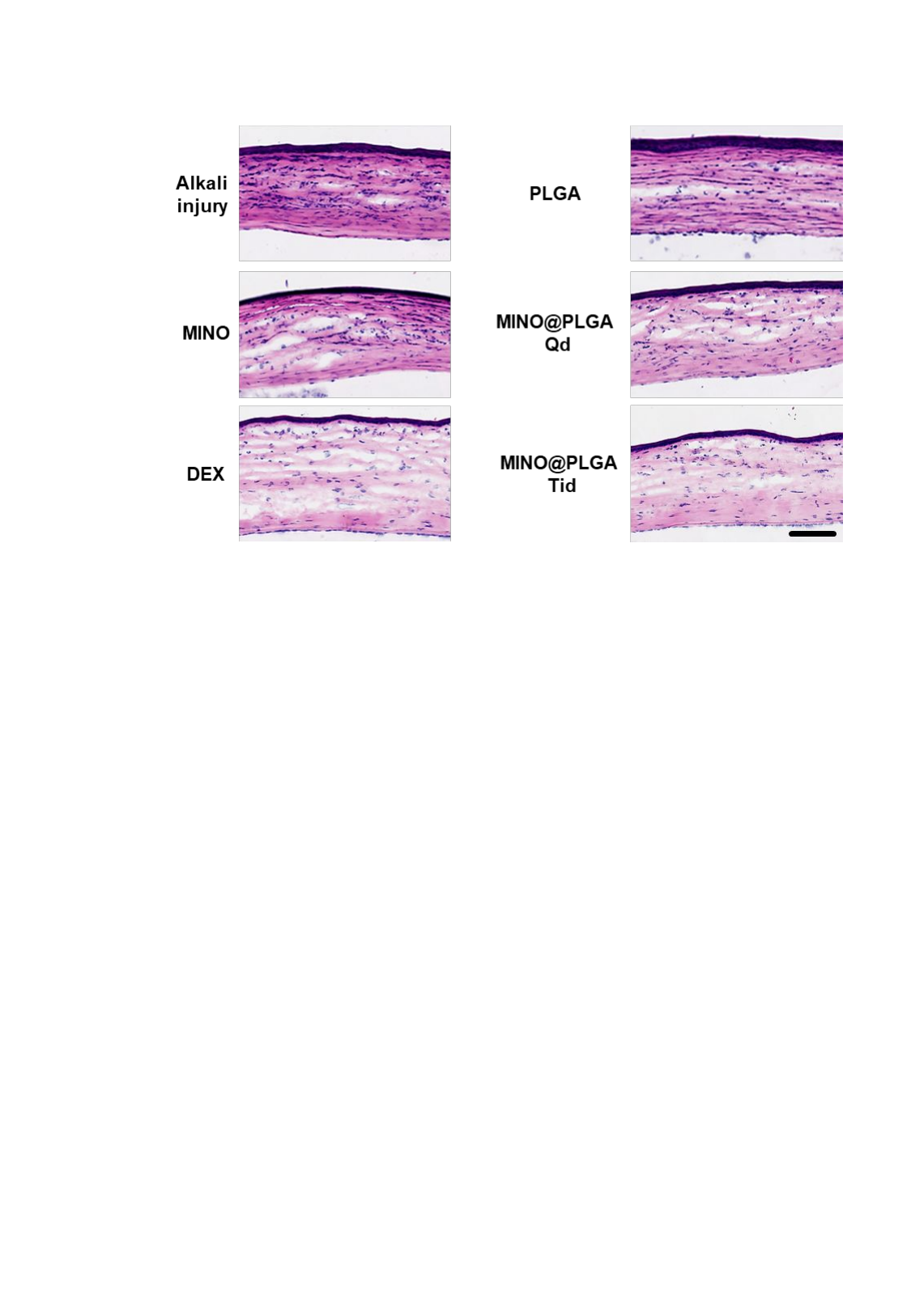

Supplement: Supplementary file 1 — Additional file 1: Figure S1. Appearance of the corneal structures by H&E. [file 12951_2024_2317_MOESM1_ESM.pptx]
